# Clinico-microbiological Profile of Septic Diabetic Foot with Special Reference to Anaerobic Infection

**DOI:** 10.7759/cureus.2252

**Published:** 2018-03-01

**Authors:** K Sasikumar, Chellappa Vijayakumar, Sadasivan Jagdish, Dharanipragada Kadambari, Nagarajan Raj Kumar, Rakhi Biswas, Subhash Chandra Parija

**Affiliations:** 1 Surgery, Jawaharlal Institute of Postgraduate Medical Education and Research (JIPMER), Puducherry, India.; 2 Microbiology, Jawaharlal Institute of Postgraduate Medical Education and Research (JIPMER), Puducherry, India.

**Keywords:** diabetic foot, limb amputation, anaerobic infection, sepsis, quality of life

## Abstract

Introduction

Diabetic foot infections are a major cause of non-traumatic amputations. The role of anaerobes in the prognosis of these infections is particularly unclear. This study was conducted with the aim of correlating microbiological profiles with clinical outcomes in these diabetic foot ulcer patients.

Methodology

This prospective observational study was done in a tertiary care centre in South India. All patients admitted with diabetic foot ulcers for two years were included in the study. Tissue biopsies were collected from the ulcer for aerobic and anaerobic cultures. The patients were grouped as those with aerobic infection alone (anaerobe negative) and those with mixed aerobic and anaerobic infections (anaerobe positive). Anaerobic culture was performed using the Robertson cooked meat (RCM) medium. The ulcer of the foot was described with respect to site, size, duration, history of previous amputation(s), and history of number and class of antibiotic intake prior to hospitalization. Clinical course and Wagner’s grades of the diabetic foot ulcers were compared for aerobic and anaerobic infections.

Results

A total of 104 patients were included in the study. There were no significant differences between the two groups with regards to duration of diabetes, random blood sugar (RBS) at the time of admission, compliance to drugs, and mode of blood sugar control and prior intake of antibiotics. Patients with anaerobic infections were found to have a higher incidence of fever in this study (38.1% vs. 14.5%; p = 0.0057), as compared to patients with aerobic infections. More than half of the patients in the anaerobic infection group presented with Wagner’s grade IV and above, as compared to the aerobic infection group (59.5% vs. 32.2%; p = 0.0059), which was statistically significant. Patients with anaerobic infections also had high numbers of major and minor amputations when compared to patients with aerobic infections.

Conclusion

Septic diabetic foot patients with fever at the time of admission and a high Wagner’s grade have a greater chance of harbouring anaerobic infections. Drugs for anaerobic coverage should be considered for wounds beyond Wagner’s grade III. Anaerobic infections resulted in increased risk of morbidity in diabetic foot ulcer patients but did not have any influence on mortality.

## Introduction

Foot infections represent a serious problem in the diabetic population. Management of this limb-threatening condition requires careful microbial isolation and appropriate antibiotic therapy [1]. As antibiotic therapy plays a vital role in the management of these ulcers, their bacterial etiology has been the focus of several studies [2]. Many studies have reported on the microbiology of diabetic foot infections over the past 25 years, but the role of anaerobes in the prognosis of these infections is particularly unclear [3]. This study was done to correlate the role of anaerobes with the clinical profiles and outcomes of patients with septic diabetic foot.

## Materials and methods

This prospective study included all patients admitted with diabetic foot ulcers for two years in a tertiary care centre in South India. Informed consent was obtained from all patients. The study was approved by the Institute Ethics Committee. Patients with diabetic cellulitis without ulcers were excluded from the study. In patients who had not been diagnosed, diabetes was defined as per the criteria used by American Diabetes Association (ADA) [3].

Baseline parameters like age, gender, duration of diabetes, method of glycemic control, and history of medical illness were noted. The ulcer of the foot was described with respect to site, size, duration, history of previous amputation(s), history of number and class of antibiotic intake prior to hospitalization, and Wagner’s grade [4]. Ulcer size was determined by multiplying the longest and widest diameters and was expressed in cm^2 ^[5]. The presence of osteomyelitis was assessed based on X-rays of the involved foot.

For microbiological studies, tissue biopsies were collected prior to the start of antibiotics. The wound was first rinsed with normal saline and then tissue biopsies were collected from deeper parts of the ulcers. Specimens were transported in a sterile bottle for aerobic studies and in Robertson cooked meat (RCM) media for anaerobic studies. The primary aerobic cultures were done in blood agar and MacConkey agar media. RCM medium and solid anaerobic media such as brain heart infusion with supplement, neomycin blood agar, and phenyl ethyl alcohol were used for isolation of anaerobes using the automated anoxamats system.

The patients were grouped as those with aerobic infections alone (anaerobe negative) and those with mixed aerobic and anaerobic infections (anaerobe positive). The clinical characteristics and the course of the diabetic foot infection in these patients were compared.

Statistical analysis

Statistical Package for the Social Sciences (SPSS for Windows, version 16.0, SPSS Inc., Chicago) was used for data analysis. Categorical variables were analyzed by chi-square test, and for continuous variables, both parametric and non-parametric tests were used. The parametric test used was student’s t test, and the non-parametric test used Mann Whitney U test. P value of <0.05 was taken as significant. 

## Results

A total of 104 patients were included in this study. Of these, 65 patients (62.5%) were male and 39 patients (37.5%) were female. The mean ages of the patients in the study groups were comparable (54.6 vs. 55.5; p = 0.50). The average duration of diabetes in the groups were comparable (5.97 vs. 6.96; p = 0.40). The mean random blood sugar (RBS; mg/dL) at the time of admission (312 vs. 299; p= 0.574) between the groups were comparable. Majority of patients (n = 55; 52.9%) were taking an oral hypoglycemic agent (OHA) as their mode of blood sugar control. Six patients (5.8%) didn’t have any form of glycemic control. There were no significant differences between the two groups with regards to duration of diabetes, RBS at the time of admission, compliance to drugs, and mode of blood sugar control and prior intake of antibiotics. Among those with anaerobic infections, seven patients (16.7%) had nephropathy, 24 patients (57%) had neuropathy, and 24 patients (57%) had retinopathy. Presence of end-organ damage was not significantly different between the groups. Patients with anaerobic infections had a higher incidence of fever at the time of admission when compared to the aerobic infection group (38.1% vs. 14.5%; p = 0.0057), which was statistically significant. Presence of leucocytosis at admission (n = 12) in each group was not statistically different between the two groups (28.6% vs.19.4%; p = 0.24) (Table 1).

**Table 1:** Base line demographic parameters in study groups N: number; DM: diabetes mellitus; RBS: random blood sugar; OHA: oral hypoglycemic agents; Rx: therapy

Demographic parameters		Anaerobe positive (n = 42)	Anaerobe negative (n = 62)	p-value
Gender (N (%))	Male	27 (64.3%)	38 (61.3%)	0.75
	Female	15 (35.7%)	24 (38.7%)	
Mean age (years)		54.6	55.5	0.50
Mean duration of DM (years)		5.97	6.96	0.40
Mean RBS (mg/dL)		312	299	0.574
Insulin + OHA (N (%))		6 (14.3%)	4 (6.5%)	0.34
Poor compliance (N (%))		20 (47.6%)	36 (58%)	0.29
Prior antibiotic Rx (N (%))		21 (50%)	28 (45.2%)	0.62
Fever (N (%))		16 (38.1%)	9 (14.5%)	0.0057
Leucocytosis (N (%))		12 (28.6%)	12 (19.4%)	0.24

**Figure 1:**
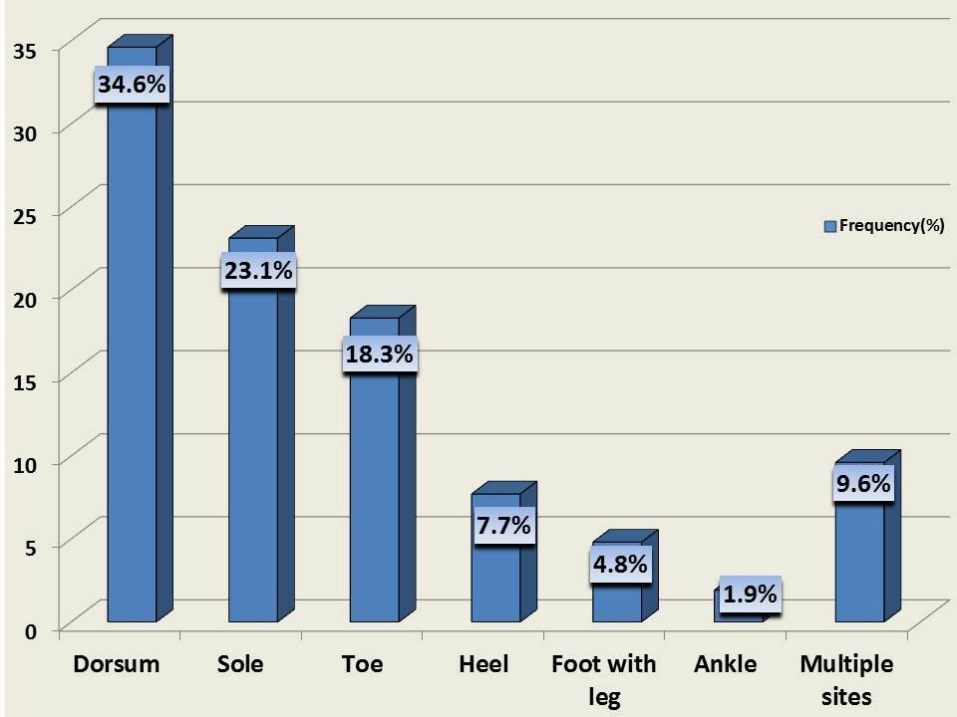
Distribution of ulcer (%) in study patients x-axis: ulcer location site; y-axis: no. of patients

The predominant site for ulcer localization was the dorsum (n = 36; 34.6%) followed by sole of the foot (n = 24; 23.1%) (Figure 1).

With respect to Wagner’s grade, a majority of patients belonged to Wagner’s grade IV (n = 41; 39.4%) followed by Wagner’s grade II (n = 33; 31.7%). Anaerobic infections were present more in Wagner’s grade IV, compared to Wagner’s grade V,  in the anaerobe positive group (21 [50%] vs. 4 [9.5%]). On comparing with the anaerobe negative group, these combined (IV & V) results were statistically significant (59.5% vs.32.2%; p= 0.0059). The difference in the mean duration of the ulcer at presentation was not significant between the two groups (18.19 vs.15; p = 0.527). The presence of osteomyelitis did not reach a significant difference between the groups (33.3% vs. 37.1%; p = 0.69). Patients with anaerobic infections also had a high number of major and minor amputations (21 [50%] vs. 23 [54.8%]), both of which attained a significant difference. Even though 18 patients (17.3%) expired in the study, the presence of anaerobic infections did not influence mortality. The mean duration of hospital stay was 34.45 days, which failed to reach any significant difference. Patient with anaerobic infections underwent desloughing at an average of 5.59 sessions. Though patients with anaerobic infections had more frequent sessions of desloughing than those with anaerobic infections at an average of 4.36 sessions, significant difference could not be achieved between the two groups. The difference in mortality rates between the groups were not significant (23.8% vs. 13.3%; p = 0.17) (Table 2).

**Table 2:** Ulcer parameters between the study groups N: Numbers

Ulcer parameters [N (%)]		Anaerobe positive (n = 42)	Anaerobe negative (n = 62)	p-value
Wagner’s grade	II	6 (14.3%)	27 (43.5%)	0.006
	IIl	11 (26.2%)	15 (24.2%)	0.81
	IV + V	25 (59.5%)	20 (32.2%)	0.0059
Vasculopathy		9 (21.5%)	8 (13%)	0.24
Osteomyelitis		14 (33.3%)	23 (37.1%)	0.69
Mean duration of ulcer (days)		18.19	14.38	0.527
Major amputations		21 (50%)	12 (19.4%)	0.00098
Minor amputations		23 (54.8%)	14 (22.6%)	0.00076
Total no. of amputation		5.59 (3.7%)	4.36 (2.8%)	0.054
Mean hospital stay (days)		34.4	26.88	0.0928
Mortality		10 (23.8%)	8 (13.3%)	0.17

A total of 170 aerobic organisms (76.23%) and 53 anaerobic organisms (23.76%) were isolated from the tissue biopsies of 104 patients. Eighty-six (82.7%) patients had poly-microbial and 18 patients (17.3%) had mono-microbial distribution of micro-organisms. All patients had a positive culture for aerobic organisms. This study found that gram-negative organisms were more predominant than gram-positive bacteria (141 [83%] vs. 29 [17%]) with regards to aerobic infection. In contrast, gram-positive anaerobes were present in greater numbers than gram-negative anaerobes [36 (67.9%) vs. 17 (32.1%)] (Table 3).

**Table 3:** Distribution of gram stain pattern in study patients N: Numbers

Type	Gram positive (n = 65)	Gram negative (n = 165)
Aerobe (N (%))	29 (17%)	141 (83%)
Anaerobe (N (%))	36 (67.9%)	17 (32.1%)

Escherichia coli was the predominant aerobic organisms isolated in culture (n = 37; 21.7%) followed by Pseudomonas aeruginosa (n = 29; 17%). Klebsiella pneumoniae and Proteus mirabilis represented 16.4% (n=28) and 11.1% (n=19) of the isolation respectively. Among gram-positive organisms, Staphylococcus aureus was isolated in 10 patients (5.8%), which formed the majority. Methicillin-resistant Staphylococcus aureus (MRSA) was isolated in only one patient (0.5%) (Table 4).

**Table 4:** Distribution of aerobic infections (%) in study patients N: Numbers; MRSA: methicillin-resistant Staphylococcus aureus

No.	Organisms (N (%))	No. of isolates (n = 170)
1.	Escherichia coli	37 (21.7%)
2.	Pseudomonas aeruginosa	29 (17%)
3.	Klebsiella pneumoniae	28 (16.4%)
4.	Proteus mirabilis	19 (11.1%)
5.	Enterococcus fecalis	11 (6.5%)
6.	Staphylococcus aureus	10 (5.8%)
7.	Streptococcus hemolyticus	2 (1.1%)
8.	MRSA	1 (0.5%)
9.	Aerobic spore bearing bacilli	1 (0.5%)
10.	Other organisms	30 (17.2%)

Amongst anaerobic infections, Peptostreptococcus species were isolated predominantly (n = 25; 47.1%), followed by Bacteroides fragilis (n = 10; 18.8%). Clostridium species were isolated in three patients only (5.4%) (Table 5).

**Table 5:** Distribution of anaerobic organisms (%) in study patients N: Numbers

No.	Organisms (N (%))	No. of isolates (n = 53)
1.	Peptostreptococcus	25 (47.1%)
2.	Bacteroides fragilis	10 (18.8%)
3.	Porphyromonas	4 (7.5%)
4.	Fusobacterium	3 (5.6%)
5.	Clostridium species	3 (5.6%)
6.	Anaerobic non-sporing bacilli	2 (3.7%)
7.	Propinobacterium	2 (3.7%)
8.	Coccobacilli	2 (3.7%)
9.	Peptostreptococcus indolincus	1 (1.8%)
10.	Peptostreptococcus anaerobicus	1 (1.8%)

**Figure 2:**
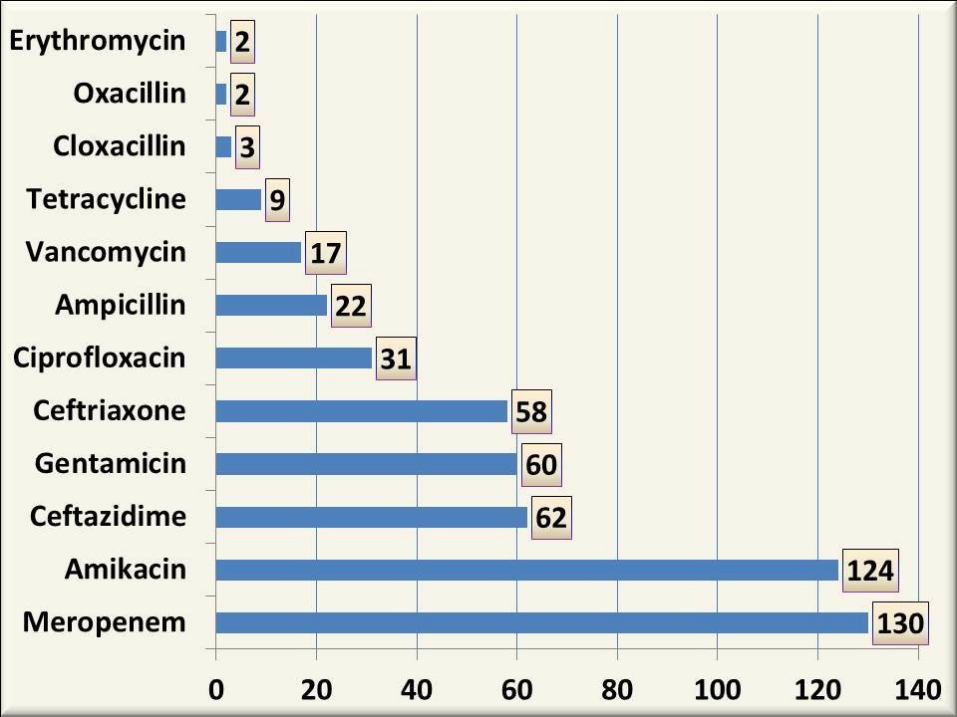
Drug sensitivity pattern of aerobes in study patients x-axis: no. of organisms; y-axis: name of the sensitive drug

Most of the aerobic organisms were sensitive to meropenem (n = 130; 76.5%) followed by amikacin (n = 124; 73%). Ceftazidime, gentamicin, and ceftriaxone had an almost equally sensitive pattern (n = 62; 36.5%, n = 60; 35.3% and n = 58; 34.1%) respectively. Thirty-one organisms (18.2%) were sensitive to ciprofloxacin and 13% of the organisms (n = 22) were sensitive to ampicillin. All Enterococcus fecalis and Staphylococcus aureus were sensitive to vancomycin. Drug sensitivity was not done for anaerobic organisms in this study (Figure 2).

## Discussion

Infected foot ulcers are a common cause of morbidity in diabetic patients, ultimately leading to dreaded complications like gangrene and amputation [4]. Management of this infection requires isolation of the microbial flora and appropriate antibiotic therapy according to sensitivity patterns [5]. This study was conducted with the aim of correlating microbiological profiles with clinical outcomes in diabetic foot ulcer patients.

Majority of patients (n = 86; 82.5%) had poly-microbial involvement, i.e., the presence of more than two different organisms. This study isolated 170 (76.23%) aerobic organisms and 53 anaerobic organisms (23.76%) from 104 cultures, with an average of 2.14 organisms per culture. This was higher compared to the findings by Shea KW et al. where cultures yielded an average 1.21 organisms [6]. In the present study, 42 patients (40%) had anaerobic infections which were higher when compared to the study conducted by Banoo S et al., where the isolation rate was 20% [7]. In both the studies, apart from tissue biopsies, pus, wound exudate, deep tissue aspirate, blister fluid etc. were collected for isolating microorganisms [6-7]. In the present study, only tissue biopsies were used as the mode of taking samples for cultures which had a greater proportion of isolating anaerobes [8].

Patients with anaerobic infection were found to have a higher incidence of fever in this study as compared to patients with aerobic infection. The presence of fever indicates systemic toxicity and deep-seated infection with extensive tissue necrosis, where the possibility of isolating the anaerobic organism in such an environment is high [9-10]. Treatment with prior antibiotic therapy did not predispose to anaerobic infection. Thirty-seven patients (35.6%) had osteomyelitis, which was diagnosed by X-rays. Of these, 14 patients (33.3%) had anaerobic infections. This was contradictory to two similar studies done by Lavery et al. and Lipsky et al., where it has been stated that anaerobic infections were less frequent pathogens in diabetic foot with osteomyelitis [11-12]. Lipsky et al. stated that more severe the infection, however, the greater the likelihood that anaerobes will be isolated [12]. In this study, out the 14 patients (33.3%) who had anaerobic osteomyelitis, six patients were in Wagner’s grade IV and one patient had Wagner’s grade V stage disease, which indicates a severe form of infection (n = 7; 50%). This could explain the high number of isolation of anaerobes when compared to the study by Lavery et al. [11].

Anaerobes were frequently isolated in patients with Wagner’s grade IV and Vl wounds. In the present study, around 60% of the ulcers (n = 25) with Wagner’s grade IV and V had anaerobic infections. This finding was concordant with other similar studies [6-7]. Patients with Wagner’s grade II diabetic foot ulcers had less anaerobes (14.3% vs. 43.%; p = 0.006) in their specimens which was statistically significant, indicating that routine use of metronidazole is not advocated in less severe forms of diabetic foot ulcers.

The study found that anaerobic infections were more common in patients undergoing amputations both minor (toe disarticulation, forefoot amputation) and major (above and below-knee amputation). Half of the patients who underwent major amputations had anaerobic infections (n = 21; 50%). This finding was in concordance with a similar study by Banoo S et al. and Moulik PK et al., where they found that anaerobes were frequently isolated in patients undergoing amputations [7, 13]. Certain bacteria like bacteroides, prevotella, and porphyromonas species are capable of expressing adhesion factors (e.g., capsular polysaccharide), tissue-damaging exoenzymes (e.g, proteases), and antiphagocytic factors (e.g., capsule, short chain fatty acids, and immunoglobulin), all of which may contribute to the impairment of wound-healing processes and also make the microorganism more invasive [14-15]. Drug sensitivity tests on anaerobic organisms could not be done due to the non-availability of test kits.

## Conclusions

Septic diabetic foot patients with fever at the time of admission and a high Wagner’s grade have a greater chance of harbouring anaerobic infections. Drugs for anaerobic coverage should be considered for wounds beyond Wagner’s grade III. Also, patients with anaerobic infections have increased risk of undergoing amputation; however, the presence of anaerobic infection did not influence mortality.

## References

[1] Abdulrazak A, Bitar ZI, Al-Shamali AA, Mobasher LA (2005). Bacteriological study of diabetic foot infections. J Diabetes Complications.

[2] Citron DM, Goldstein EJ, Merriam CV, Lipsky BA, Abramson MA (2007). Bacteriology of moderate-to-severe diabetic foot infections and in vitro activity of antimicrobial agents. JCM.

[3] The Expert Committee on the Diagnosis and Classification of Diabetes Mellitus (2003). Report of the expert committee on the diagnosis and classification of diabetes mellitus. Diabetes Care.

[4] Frykberg RG (2002). Diabetic foot ulcers: pathogenesis and management. Am Fam Physician.

[5] Gadepalli R, Dhawan B, Sreenivas V, Kapil A, Ammini AC, Chaudhry R (2006). A clinico-microbiological study of diabetic foot ulcers in an Indian tertiary care hospital. Diabetes Care.

[6] Shea KW (1999). Antimicrobial therapy for diabetic foot infections. A practical approach. Postgrad Med.

[7] Banoo S, Shubha DS, Shashidhar V, Venkatesha D (2012). Bacterial and clinical profile of diabetic foot patients. Ann Trop Med Public Health.

[8] Hokkam EN (2009). Assessment of risk factors in diabetic foot ulceration and their impact on the outcome of the disease. Prim Care Diabetes.

[9] Lipsky BA (2004). Medical treatment of diabetic foot infections. Clin Infect Dis.

[10] Landis SJ (2008). Chronic wound infection and antimicrobial use. Adv Skin Wound Care.

[11] Lavery LA, Sariaya M, Ashry H, Harkless LB (1995). Microbiology of osteomyelitis in diabetic foot infections. J Foot Ankle Surg.

[12] Lipsky BA, Berendt AR (2000). Principles and practice of antibiotic therapy of diabetic foot infections. Diabetes Metab Res Rev.

[13] Moulik PK, Mtonga R, Gill GV (2003). Amputation and mortality in new-onset diabetic foot ulcers stratified by etiology. Diabetes Care.

[14] Maiti PK, Haldar J, Mukherjee P, Dey R (2013). Anaerobic culture on growth efficient bi-layered culture plate in a modified candle jar using a rapid and slow combustion system. Indian J Med Microbiol.

[15] Haldar J, Mukherjee P, Mukhopadhyay S, Maiti PK (2017). Isolation of bacteria from diabetic foot ulcers with special reference to anaerobe isolation by simple two-step combustion technique in candle jar. Indian J Med Res.

